# Anti-Dengue Sanitation Practices: A Health Education Approach for Municipal Sanitary Workers in Puducherry, India

**DOI:** 10.7759/cureus.65227

**Published:** 2024-07-23

**Authors:** Priskilla Johnson Jency, Kozhithodi Eranthodi Rishla, Muhammed M Jabir, Balakrishnan Vijayakumar, Raja Jeyapal Dinesh, Rajendran Dhanalakshmi

**Affiliations:** 1 Epidemiology and Operational Research, Indian Council of Medical Research-Vector Control Research Centre, Puducherry, IND; 2 Biostatistics and Vector-Borne Disease Modelling, Indian Council of Medical Research-Vector Control Research Centre, Puducherry, IND

**Keywords:** vector control, sanitary workers, information literacy, health education, dengue

## Abstract

Introduction

Dengue is a mosquito-borne disease of global health concern, especially in tropical areas. *Aedes aegypti*, its vector, thrives in inadequate sanitation conditions. The role of sanitary workers is pivotal in dengue control and prevention efforts; hence, educating them is essential for enhancing their vector control awareness.

Methods

This study was conducted among 109 municipal sanitary workers in selected areas of Puducherry, India. Their baseline knowledge, attitudes, and practices (KAP) regarding dengue were assessed through a pretested, semi-structured questionnaire, followed by a targeted health education intervention incorporating novel communication methods such as pocket awareness cards. The impact of the intervention was assessed through an increment in KAP scores, qualitative interviews, and surprise visits to the field during their work hours.

Results

The mean (± SD) scores in terms of knowledge (4.29 ± 1.77 vs. 7.17 ± 1.02; p < 0.01), attitudes (3.58 ± 1.42 vs. 4.69 ± 0.71; p < 0.01), and practices (1.98 ± 0.84 vs. 4.28 ± 1.12; p < 0.01) significantly increased post-intervention. Qualitative interviews revealed the utility of the intervention, with additional insights on implementation barriers and strategies for the future.

Conclusion

The study’s findings imply that the targeted health education intervention for sanitary workers was effective in improving their knowledge and practices on dengue control. These results demonstrate the potential of future educational initiatives to promote vector control measures among sanitary staff and thereby combat dengue transmission in the community.

## Introduction

Dengue fever is the most rapidly spreading viral infection transmitted by mosquitoes, posing a serious threat to public health. With climate change, its rise has been particularly evident in tropical and subtropical areas. Dengue fever is present in more than 100 countries across WHO regions, with Asia representing over 70% of the global burden [1]. An alarming 5.2 million cases of dengue were officially reported to the WHO in 2019, compared to ≈5 lakh cases in 2000. With 100-400 million infections reported every year, the disease currently poses a significant hazard to over half of the world’s population [1].

The National Center for Vector Borne Diseases Control in India had registered a total of 63,280 dengue cases as of September 30, 2022 [2]. Dengue positivity in India surges during the monsoon and post-rainy seasons, and people of younger age groups are particularly vulnerable. Higher seroprevalence rates, cocirculation of multiple serotypes, and the occurrence of secondary infections may all contribute to an increased risk of severe dengue infections in India [3]. Once confined to urban regions, the disease has now spread to semi-urban and rural areas. This heightened prevalence is characterized by a number of interrelated variables, such as unrestrained urban sprawl, population growth, and improper waste management practices, which create an ideal breeding ground for mosquitoes [4]. A study in Brazil discovered favorable breeding places in household garbage, emphasizing the critical need for prioritizing appropriate garbage disposal and packing of solid wastes [5].

Even in our study area, Puducherry, a concentration of dengue cases has been attributed to inadequate environmental sanitation. Breeding sources were noted in vacant plots and open, unmanned areas, which were primarily used as waste disposal sites [6]. Also, the occurrence of dengue and dengue hemorrhagic fever is intricately linked to environmental conditions that facilitate the presence of breeding sites for the dengue vector, the *Aedes aegypti *mosquito. Hence, there is a pressing requirement to prioritize environmental sanitation for vector control.

The significant role of sanitary workers in waste disposal is instrumental in directly combating dengue. Sanitary workers are individuals who clean, maintain, empty, or operate sanitation technologies at every stage of the sanitation chain [7]. Through their routine waste management services, they help to prevent and control several vector-borne diseases, particularly dengue. They frequently serve as a link between communities and municipal services, providing a unique opportunity for community engagement in dengue prevention. Furthermore, sanitary workers are frequently exposed to vector-borne diseases such as dengue, chikungunya, and malaria [8]. Despite their crucial role in dengue protection, there is limited research that examines sanitary workers’ contributions and analyzes their knowledge, attitude, and actions about dengue prevention. Given the current shortcomings in environmental sanitation and the potential health risks faced by sanitary workers, it would be beneficial to equip them with the knowledge and tools needed to boost their impact on public health while also improving their own safety against vector-borne diseases [8]. Hence, the primary objective of this research was to design and test a health education strategy to train sanitary personnel in dengue prevention and vector control.

## Materials and methods

Study setting

The study was conducted in Puducherry, a coastal town in south India. Puducherry is hot and humid, with a temperature range of 26 to 38 °C [9]. The Local Administration Department (LAD) of Puducherry operates via municipalities in urban areas and commune panchayats in rural areas. For administrative purposes, the municipalities are further subdivided into blocks and wards. The sanitation works are carried out by both municipal sanitary workers and laborers employed under the Swachhata Corporation. They are overseen by municipal supervisors [10].

Study design and period

**Figure 1:**
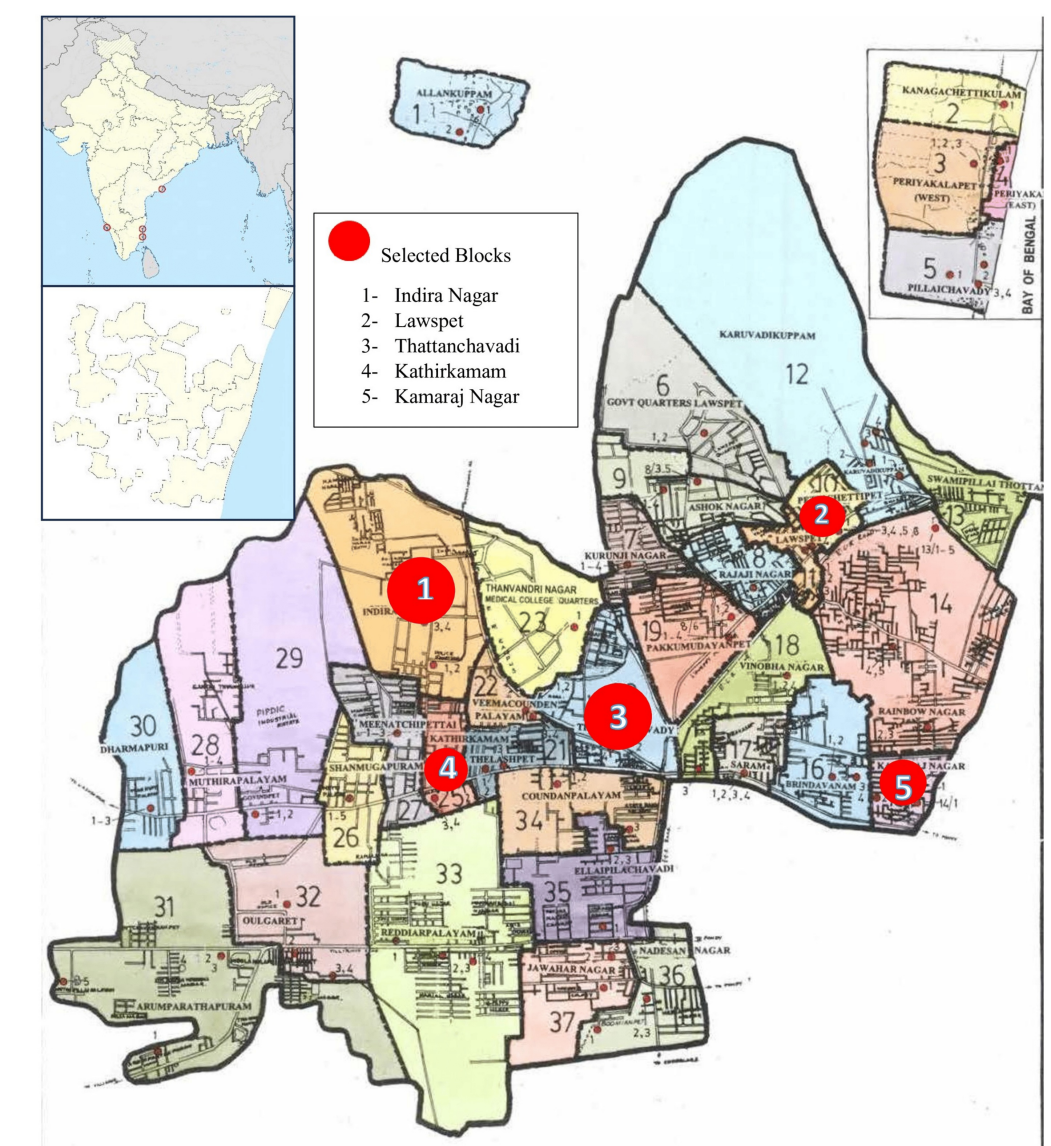
Map showing areas of intervention among sanitary workers Image adapted from the website of Oulgaret Municipality, Government of Puducherry [11]

This quasi-experimental (pre-test and post-test) study was carried out in three stages spanning from February to July 2023 among the sanitary workers in five selected blocks of the Oulgaret municipality in Puducherry (Figure 1).

Sample size

Assuming a baseline knowledge of the study population on dengue of 30% and an anticipated proportion of knowledge after the intervention of 50%, with a 5% margin of error, a 95% confidence interval, and a non-response rate of 20%, the minimum sample size was calculated as 115 (applying McNemar’s test).

Sampling method

Out of the seven blocks of Oulgaret municipality, the study focused on five blocks, namely Indira Nagar, Lawspet, Thattanchavady, Kathirkamam, and Kamaraj Nagar. The remaining two blocks were excluded due to specific challenges, such as a lack of permanent employees and other logistical issues. Participants were enrolled through a simple random sampling process in each of these five blocks. The inclusion criteria for participation were sanitary workers who worked under permanent employment for more than one year and who provided consent. Those on working contractual terms and those who took extended leave during the study period were excluded.

Study procedure

The study was conducted in three phases.

Phase 1: Baseline Assessment

A pre-tested, semi-structured questionnaire was employed to evaluate the existing knowledge pertaining to dengue fever, its vectors, and preventive measures. The questionnaire was prepared based on available literature [11]. It encompassed four main domains: (1) sociodemographic details, (2) knowledge, (3) attitude, and (4) practices pertaining to dengue. The questions were pilot-tested among sanitary workers (n = 20) from blocks other than the study area and were further refined based on expert opinion prior to their use. Each right answer was scored 1, and the wrong answer was scored 0. For questions with multiple responses, a score of 1 was given if more than one answer was correct. The original Bloom’s cutoff points, 18, were used to classify the scores into three levels: good (80-100%), moderate (60-79%), and poor (less than 60%). Further qualitative interviews using an interview guide were conducted among sanitary inspectors to obtain their insights on dengue-based health education activities.

Phase 2: The Health Education Intervention

**Figure 2:**
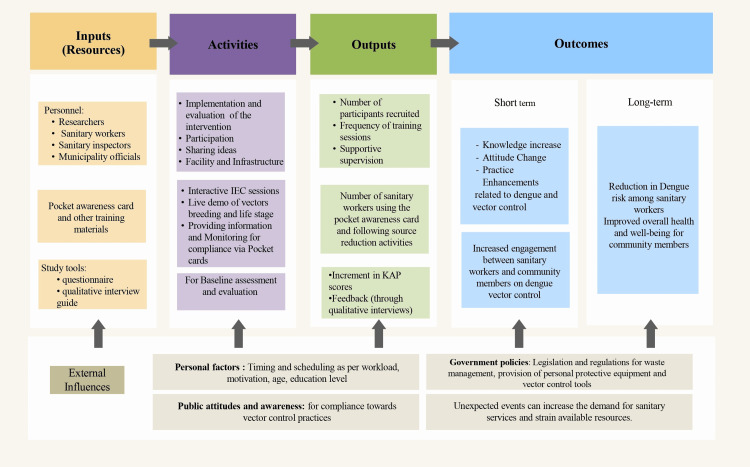
Logical model of anti-dengue sanitary practices intervention

After a thorough assessment of the study participants’ current level of understanding and needs regarding dengue, a logical model for the health education intervention was curated (Figure 2). Accordingly, training sessions were conducted, emphasizing vector bionomics, life cycle, and symptoms of dengue fever. Live demonstrations of all four life stages of *Aedes *vectors and breeding sites in their locality were carried out. The breeding sites were categorized into four types for better identification during their fieldwork.

To ensure compliance, another communication tool was designed for the study, i.e., a pocket awareness card in the local language, Tamil. The card had step-by-step information on dengue fever, vectors, symptoms, and preventive measures. As most study participants were illiterate, pictures were predominantly used. On the back side of the card, there was an observation sheet designed for practicing source reduction at least twice a week. The implementation of this practice was overseen by the respective municipal supervisors. The municipal supervisors were trained to oversee the workers prior to the intervention.

Phase 3: Post-Intervention Assessment

The impact of the intervention was assessed using the same semi-structured questionnaire used in Phase 1. Compliance with practices was ensured through surprise field visits by the researchers. Further, the experiences of sanitary workers during the intervention were recorded through anonymous feedback forms and qualitative interviews.

Data analysis

For quantitative analysis, data were entered in Microsoft Excel (Microsoft Corporation, Redmond, Washington, United States), and analysis was performed using STATA 14.2 for Windows (StataCorp. 2023, Stata Statistical Software: Release, StataCorp LLC, College Station, Texas, United States). Data were expressed as mean and SD for continuous variables and frequency with percentage (%) for categorical variables. A paired t-test was used to estimate the difference between pre- and post-intervention scores. An independent t-test (Student’s t-test) was used to test the difference between groups or categories. A p-value less than 0.05 was considered statistically significant. The qualitative data collected after the intervention were transcribed into English. The responses were analyzed using a grounded theory approach using ATLAS.ti (version 9.0). The coding process involved reading through the interview transcripts and open coding portions of the text. These open codes were then categorized into axes that were part of the core category.

Ethical considerations

Ethical clearance was obtained from the Institutional Human Ethics Committee before initiating the study (certificate no.: VCRCIHEC-0823/N/A). Approval for the research was also secured from the LAD of Puducherry. Informed written consent was obtained from all participants. The privacy and confidentiality of the responses received were ensured. Participants had the right to withdraw from the study at any point in time and were given the freedom not to answer any question with which they felt uncomfortable. Every step of this study adhered to the relevant guidelines and precepts stated in the Declaration of Helsinki.

## Results

Initially, 115 sanitary workers were included in the baseline survey. However, during the post-assessment phase, six of them were lost to follow-up (prolonged leave for personal reasons; not reachable on the phone). Thus, only 109 responses were included for analysis.

Sociodemographic characteristics

Table 1 provides the sociodemographic information of the study participants. The mean age of the study participants was 48.8 ± 8.7 years, with a predominance of female participants (104; 95.4%), as women were primarily responsible for routine waste management activities while men were more engaged in transportation. Out of the 109 participants, 5 (3.7%) of them reported experiencing a dengue infection in the past.

**Table 1:** Sociodemographic characteristics of the study population (n = 109) The data has been represented as numbers (n) and percentages (%) for a total of 109 participants.

Distribution of respondents	Category	Number (%)
Age	≤45 years	29 (26.6)
	45-55 years	60 (55.1)
	≥55 years	20 (18.3)
Gender	Male	5 (4.6)
	Female	104 (95.4)
Educational status	Illiterate	82 (75.2)
	Primary	14 (12.8)
	High school	13 (11.9)
Area of work	Indira Nagar	18 (16.5)
	Lawspet	10 (9.2)
	Thattanchavadi	20 (18.3)
	Kathirkamam	37 (33.9)
	Kamaraj Nagar	24 (22.0)
Years of experience	≤7 years	45 (41.3)
	>7 years	64 (58.7)

Knowledge, Attitude, and Practices

Table 2 shows that, compared to the baseline survey, there was an improvement in the knowledge of the study participants (n = 109) after the intervention. A total of 107 (98.2%) of the study participants were aware that dengue is transmitted through mosquito bites, whereas in pre-intervention, only 53 (48.6%) knew it. One hundred (91.7%) participants were aware of the common symptom of dengue after intervention, as opposed to 72 (67%) of them before intervention. Moreover, 84 (77.1%) participants responded *Aedes *mosquitoes as dengue vectors, whereas in the pre-intervention period, only 10 (9.2%) said so. Eighty (73.4%) of them had knowledge about *Aedes *biting habits in the post-intervention period, compared to 11 (10.1%) in the pre-intervention period. Significant improvement was also noted in the knowledge regarding breeding sites and anti-dengue practices (Table 2).

**Table 2:** Participants’ knowledge, attitude, and practices toward dengue (n = 109) The data has been represented as numbers (n) and percentages (%) for a total of 109 participants.

Questions	Pre-intervention	Post-intervention
	Number (%)	Number (%)
Knowledge		
Mode of transmission		
Mosquito bite	53 (48.5)	107 (98.2)
Contaminated food/water	10 (9.2)	1 (0.9)
Don’t know	46 (42.3)	1 (0.9)
Common symptoms		
Fever and headache	72 (66.1)	100 (91.7)
Nausea and vomiting	25 (22.9)	32 (29.4)
Rashes	16 (14.7)	37 (33.9)
Muscle and joint pain	19 (17.4)	56 (51.4)
Period of dengue incidence		
Pre-monsoon	20 (18.3)	6 (5.5)
Post-monsoon	73 (67.0)	100 (91.7)
Don’t know	16 (14.7)	3 (2.8)
Knowledge of Aedes mosquitoes		
Yes	10 (9.2)	84 (77.1)
No	99 (90.8)	25 (22.9)
Biting time		
Day time	11 (10.1)	80 (73.4)
Night	56 (51.4)	6 (5.5)
Don’t know	42 (38.5)	23 (21.1)
Breeding sites		
Stagnant water	80 (73.4)	89 (81.7)
Septic tank	11 (10.1)	20 (18.4)
Drains and garbage	20 (18.4)	22 (20.2)
Clean water-holding containers	7 (6.4)	64 (58.7)
Mud pools	48 (44.0)	0 (0)
Reasons for dengue spread		
Dumping in vacant sites/drainage	80 (73.4)	102 (93.6)
Not using personal protection measures	29 (26.6)	35 (32.1)
Specific measures to control dengue		
Chemical spray	69 (63.3)	75 (68.8)
Source reduction	27 (24.8)	71 (65.1)
Community awareness	19 (8.3)	70 (64.2)
Case reporting	4 (3.7)	4 (3.6)
Attitude		
Risk of getting dengue	59 (54.1)	88 (80.7)
Dengue fever can be preventable	65 (59.6)	105 (69.3)
Community participation lowers the risk	87 (79.8)	106 (97.3)
Environmental sanitation plays a role in dengue control	83 (76.2)	106 (97.3)
Willing to take part in a public activity for dengue control	97 (88.9)	107 (98.2)
Practices		
Frequency of source reduction		
Always	16 (14.7)	73 (67.0)
Sometimes	67 (61.5)	36 (33.0)
Never	26 (23.9)	0 (0)
Personal protective measures during work		
Insect repellent	10 (9.2)	65 (59.7)
Long-sleeved dress	10 (9.2)	67 (61.5)
Oil application on the skin	1 (1.0)	47 (43.1)
Gloves and mask	47 (43.1)	20 (18.4)
No preventive measures	41 (37.6)	9 (8.3)
Treatment-seeking behavior		
Self-treatment	15 (13.8)	3 (2.8)
I will go to the hospital	94 (86.2)	106 (97.3)

In terms of a general attitude toward dengue and preventive measures, 59 (54.1%) participants perceived the risk of getting dengue, which improved to 88 (80.7%) following the intervention. Similarly, there was a notable change in attitude toward prevention (59.6%, n = 65 vs. 69.3%, n = 105), the role of community participation and environmental sanitation (79.8%, n = 87 vs. 97.3%, n = 106), and positive interest toward public activities (88.9%, n = 97 vs. 98.2%, n = 107).

Regarding dengue-related practices, 73 (67%) of workers mentioned having undertaken source reduction activities, as opposed to 16 (14.7%) before the intervention. They also reported improvements in their personal protection measures and treatment-seeking behavior, as summarized in Table 2.

Increment in Scores

Table 3 shows a significant increment in scores across age, education, and work experience categories after the intervention. Further, independent-t-test analyses revealed that participants aged 50 years or younger (p = 0.03) and those educated to a minimum of primary school level (p = 0.02) are found to have acquired more knowledge than their counterparts. Education status was also found to influence attitude change (p = 0.04). However, no such influences are found in adopting practices after the intervention.

**Table 3:** Increment in participants’ scores after the intervention (n = 109) The pre-intervention and post-intervention scores are expressed as mean ± SD. p-values less than 0.05 are statistically significant.

Scores (mean ± SD)		Knowledge			Attitude			Practice		
Categories	n	Pre-intervention	Post-intervention	p-value*	Pre-intervention	Post-intervention	p-value*	Pre-intervention	Post-intervention	p-value*
Age										
50 years and below	63	4.34 ± 1.75	7.34 ± 0.84	<0.001	3.60 ± 1.37	4.7 ± 0.42	<0.001	1.95 ± 0.85	4.20 ± 1.10	<0.001
More than 50 years	46	4.21 ± 1.81	6.93 ± 1.18	<0.001	3.56 ± 1.50	4.60 ± 0.97	<0.001	2.02 ± 0.85	4.39 ± 1.14	<0.001
Education status										
Illiterate	82	4.13 ± 1.81	7.04 ± 1.07	<0.001	3.42 ± 1.47	4.66 ± 0.78	<0.001	1.98 ± 0.85	4.31 ± 1.07	<0.001
Literate	27	4.78 ± 1.58	7.56 ± 0.70	<0.001	4.07 ± 1.14	4.81 ± 0.40	0.003	1.96 ± 0.85	4.18 ±1.22	<0.001
Work experience										
Seven years and below	45	4.24 ± 1.81	7.35 ± 0.74	<0.001	3.82 ± 1.30	4.80 ± 0.40	<0.001	1.91 ± 0.87	4.28 ± 0.99	<0.001
More than seven years	64	4.32 ± 1.7	7.04 ± 1.16	<0.001	3.42 ± 1.48	4.62 ± 0.86	<0.001	2.03 ± 0.83	4.20 ± 1.21	<0.001

Change as per Bloom’s Cutoff Point

**Figure 3:**
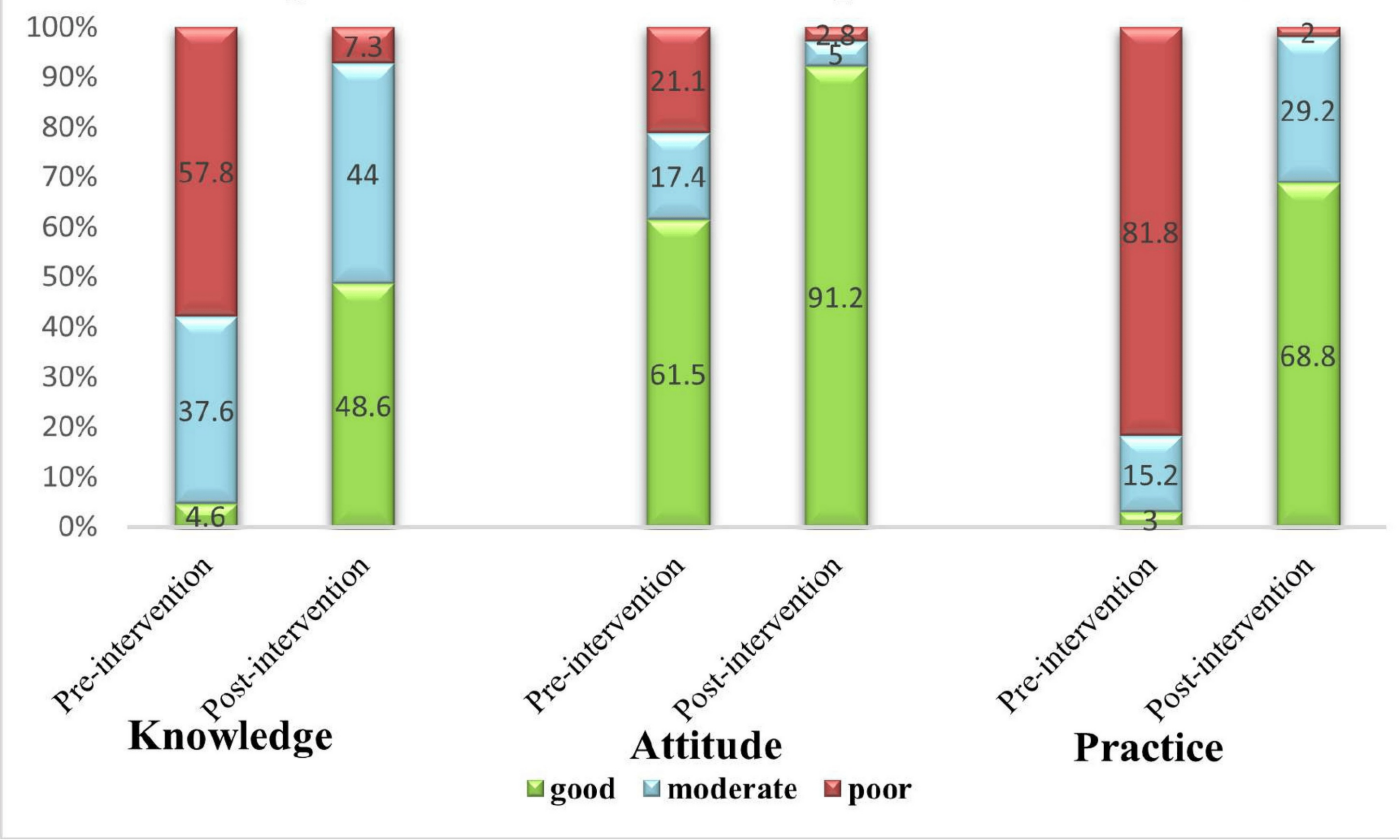
Change due to intervention (as per Bloom’s cutoff)

Figure 3 shows the participants’ status before and after the intervention, which is categorized according to Bloom’s cutoff point. The “poor knowledge level” category saw a substantial decline to just eight (7.3%) of participants, while the “good knowledge level” category experienced a remarkable increase, with 53 (48.6%) of the participants. The level of attitude has significantly improved after the intervention. A total of 106 (91.2%) of the participants had good attitude levels, and only three (2.8%) of them retained poor attitude levels after the intervention. Moreover, 75 (68.8%) of the participants had a good practice score post-intervention.

Findings from a qualitative study

**Figure 4:**
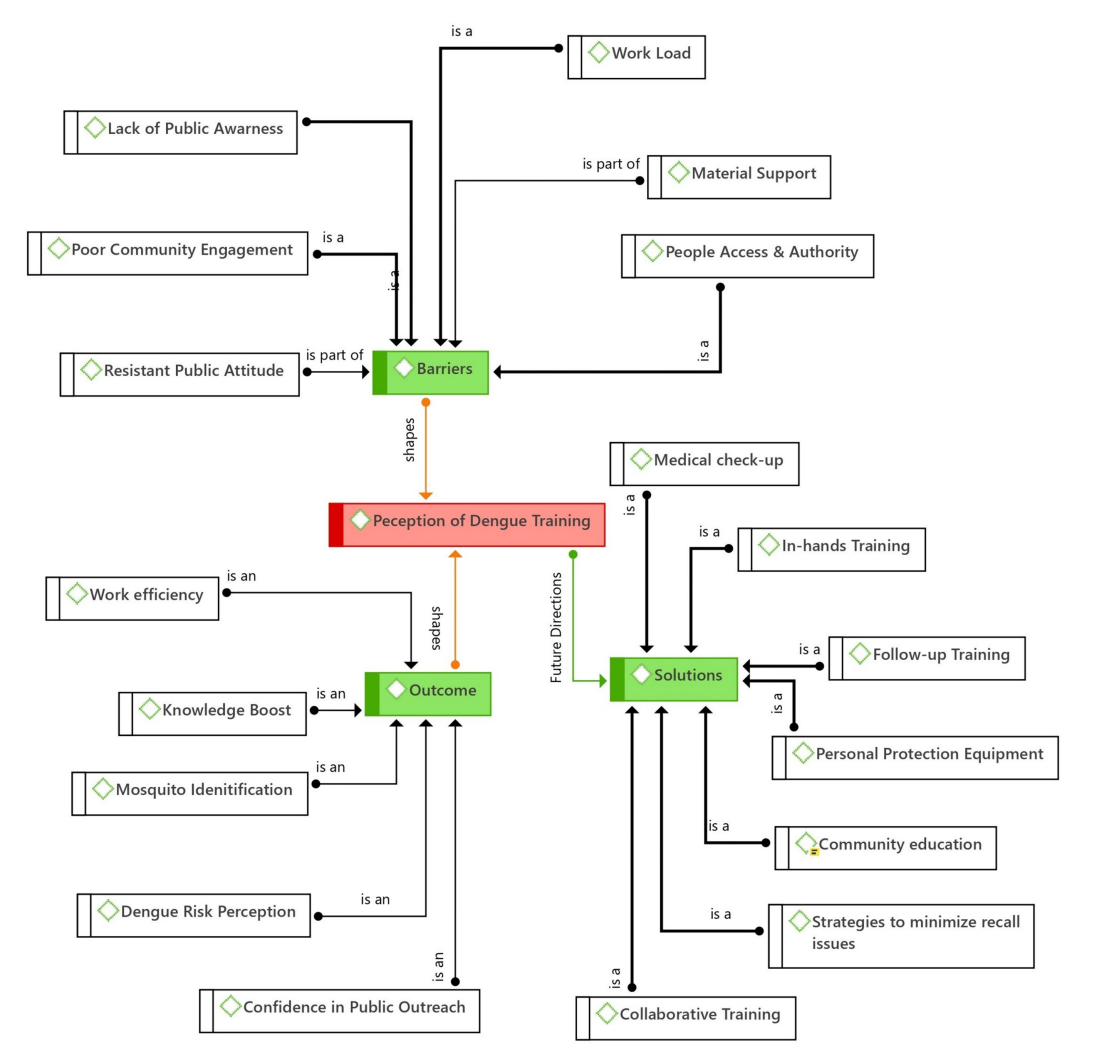
Network diagram depicting sanitary workers’ perceptions of the intervention

The primary aim of the qualitative phase of this study was to gain insight into participants’ perceptions of the health education intervention. The qualitative data obtained through focus group discussions with sanitary workers underwent thematic analysis to understand their views on the training. The qualitative network diagram, as shown in Figure 4, illustrates the main codes and categories that emerged from the analysis. The diagram is structured around the central theme “perception on dengue vector control training” and three distinct categories, including “outcome,” “barriers,” and “solutions.” The first segment highlights positive outcomes reported by the participants regarding training, the second outlines barriers to the practical application of the learning, and the final category presents the recommendations made by sanitary workers to enhance training effectiveness and address identified implementation challenges.

Outcome

The respondents unanimously reported that the training has enhanced their understanding of dengue disease, vectors, and control methods. It has also led to increased work efficiency on source reduction activities and equipped them with the skills to accurately identify the dengue-transmitting mosquito *A. aegypti*. A total of 93 out of 109 participants acknowledged that the dengue pocket card improved their understanding of dengue and its preventive measures. Following the training, they have felt a sense of self-confidence in engaging with the public to educate them regarding dengue prevention measures. They also expressed that the training has fostered a sense of self-risk perception of dengue fever and made them aware of their vulnerability to contracting the disease in order to adopt necessary precautions and safety measures.

The card and training program were very useful for me. Previously, I was not aware of dengue that much. I feel confident in educating the public now. Now I can identify breeding sources, and I am also doing proper source reduction - Sanitary worker, Block 2

Now I am aware of the importance of removing water stagnation. During my work, I have observed some stages of mosquitoes that you people showed on that day, and I immediately discarded - Sanitary worker, Block 4

Barriers

The participants encountered a spectrum of challenges, each posing significant barriers to implementing the learnings in their daily practices. Poor community engagement emerged as a central hurdle owing to a lack of awareness among the public and a resistant attitude. This derailed the sanitary workers’ efforts to reduce breeding sites. Compounding this, the participants also reported that excessive workloads, a lack of manpower, and material support could potentially compromise the quality of implementation.

People are not cooperating. Even though we are cleaning everywhere, they are throwing garbage indiscriminately - Sanitary worker, Block 4

We have so much work pressure. I tried to educate people and stick to frequent source reduction, but sometimes due to workload, I would give up - Sanitary worker, Block 5

The attitude of some households was really bad. They threw garbage at the next house in order to keep their area clean - Sanitary worker, Block 2

Solutions

Most of the respondents in this study expressed that regular follow-up training is required to reinforce their knowledge and skills regarding dengue disease control. They suggested hands-on and collaborative training involving other key players, such as accredited social health activist workers and Anganwadi workers, to create a comprehensive and coordinated approach to dengue control. Implementing routine medical checkups to detect dengue infection was emphasized by sanitary workers to safeguard their health against vector-borne diseases. They felt equipping them with necessary personal protective equipment against mosquito bites during sanitation work could significantly reduce the risk of contracting dengue illness. To overcome the implementation challenges and foster active community participation, the participants have highlighted the need for community education initiatives regarding dengue disease control.

Community participation needs to be improved. People are not cooperating well. That again creates breeding sites - Sanitary worker, Block 3

If we get medical checkups and personal protective equipment, it will be more useful for us. We have been working in the same place for hours. So, the risk of getting dengue fever is quite high - Sanitary worker, Block 5

Recall issues among us should be addressed in future sessions - Sanitary worker, Block 1

## Discussion

The present study is one of the first, highlighting the importance of training sanitary workers, an important stakeholder in dengue vector control in India. Prior to the intervention, notable deficiencies were evident in the study population’s understanding of dengue symptoms, preventive measures, and the behaviors of *Aedes *mosquitoes in terms of biting and breeding. Out of 109 participants, only 53 (48.6%) were aware that dengue is a mosquito-borne disease, a finding similar to a study from Indonesia [12]. Accordingly, 72 (66.1%) of the participants reported fever and headache, and 19 (17.4%) reported muscle or joint pain as probable symptoms of dengue fever, which were similar to a study from Delhi. A total of 11 (10.1%) opined that dengue vectors bite in the daytime only, which was contrary to a study from Delhi before intervention [13].

After the intervention, 107 (98.2%) of them were aware of the mode of transmission of dengue, similar to a study from India. The extent of improvement in knowledge on dengue fever as indicated in this research is similar to the observations from analogous studies carried out in countries like India, Pakistan, and Jamaica [14-16]. A positive shift was also exhibited in the attitudes of the sanitary workers toward dengue prevention. A total of 88 (80.7%) were aware of the risk of getting dengue, 105 (69.3%) responded that dengue fever can be prevented, and 107 (97.3%) responded that community participation and environmental sanitation will help to control dengue, corroborating with other studies [17]. The willingness to participate in public campaigns also increased post-intervention, according to a study in Sri Lanka [18]. Practices like treatment-seeking behavior and frequency of source reduction were increased. Insect repellents were used by 65 (59.6%), and 67 (61.5%) wore long-sleeved dresses as personal protective measures to get rid of mosquito bites after the intervention, which was similar to a study from North India [19]. The observations under the practice domain might be due to a momentary increase in awareness of the risk of contracting dengue and following personal protection during their working hours. However, other barriers to practice should be addressed to ensure a sustained behavioral change [4].

The analysis of the qualitative data has revealed valuable insights into the intervention. In India, traditional approaches to health education involve the use of posters, banners, presentations, and audio/video messages [20]. The positive feedback from the participants reflects the strength of the innovative methods used in this study and also underscores the value of similar educational tools in public health campaigns for addressing vector-borne diseases. During the discussion with participants, a notable obstacle hindering the effective implementation of vector control was the lack of community participation and a resistant attitude from the public. This finding aligns with previous studies conducted in India, highlighting the difficulty of engaging communities [21]. Despite evidence indicating that community behavior significantly influences vector abundance and its control [22] and that the success of vector control relies on community awareness and engagement [4,21], there continues to be inadequate participation from affected communities in these efforts. Participants also highlighted the necessity of continued training to ensure that knowledge is retained and effectively translated into consistent action. This argument is valid, as many reports indicate that the sanitary workers in India engaged in high-risk work such as cleaning sewers and septic tanks need continuous training and education [22]. Their concerns about the lack of protective gear and medical assistance are appropriate, given that many of them engage in risky tasks in their daily routine. The same has been highlighted in literature and reports, emphasizing the unsafe work environment without adequate protective measures in India [23]. Despite guidelines provided in the Swachh Bharat Mission for the safety of sanitary workers [24], the agencies implementing these rules at the grass-roots level are not adhering to them, leaving workers to perform their duties without adequate protection. This underscores the need for increased attention to the safety and well-being of sanitary workers in India, especially from vector-borne diseases.

Strengths and limitations

This health education-based interventional study on dengue and vector control among sanitary workers exhibited several notable strengths that contributed to the robustness of its findings. One of the key strengths is its targeted approach, specifically tailoring the intervention to a critical group directly involved in dengue prevention efforts. Furthermore, the study employs a mixed-methods approach for a comprehensive evaluation of the impact of the intervention on multiple dimensions, enhancing the validity and depth of the results. Moreover, the engagement of sanitary workers in completing associated worksheets showcases a practical application of learned knowledge, potentially reinforcing behavior change.

This study presents notable limitations that must be considered while interpreting its findings. Firstly, the relatively short duration of the study may have hindered our ability to capture any longer-term effects or potential shifts in the behavior of sanitary workers. The utilization of a pre-post-study design without a control group also hinders establishing a clear link between the intervention and changes in knowledge, attitudes, and practices. Social desirability bias also warrants consideration, as participants may have felt inclined to provide socially acceptable responses post-intervention.

## Conclusions

The study tried to identify the main issues that prevent sanitary workers from achieving sustainable vector control, namely inadequate dengue training programs, poor attitudes and practices toward dengue, and a lack of material support and human resources. The improvement observed in their understanding of dengue, attitude, and practices toward preventive measures signifies the intervention's efficacy in disseminating valuable information. Reducing barriers and focusing on the cues to action will enhance their performance. Also, through the collaborative efforts of the health department, community members, and other stakeholders, sustained vector control can be achieved in the study areas. Robust research is required to develop targeted behavioral change communication interventions for dengue vector control among sanitary workers to further strengthen the causality argument of this study.
